# Validating a generic cancer consumer quality index in eight European countries, patient reported experiences and the influence of cultural differences

**DOI:** 10.1186/s12885-021-07943-0

**Published:** 2021-03-06

**Authors:** A. Wind, E. D. Hartman, R. R. J. P. Van Eekeren, R. P. W. F. Wijn, J. Halámková, J. Mattson, S. Siesling, W. H. van Harten

**Affiliations:** 1 Rijnstate Hospital, Arnhem, The Netherlands; 2 Department of Health Technology and Services Research, technical Medical Centre, Faculty of behavioural, management and social sciences (BMS), University of Twente, Enschede, The Netherlands; 3 Departement of Urology, Jeroen Bosch Hospital, Den Bosch, The Netherlands; 4 Department of Comprehensive Cancer Care, Masaryk Memorial Cancer Institute, Brno, Czech Republic; 5 Department of Comprehensive Cancer Care, Faculty of Medicine, Masaryk University, Brno, Czech Republic; 6 Helsinki University Hospital Comprehensive Cancer Center, Helsinki, Finland; 7 Department of Research and Development, Netherlands Comprehensive Cancer Organisation (IKNL), Utrecht, The Netherlands

**Keywords:** Consumer quality, Patient satisfaction, Cancer care, Cultural dimensions

## Abstract

**Background:**

Taking patient centeredness into account is important in healthcare. The European Cancer Consumer Quality Index (ECCQI) is a validated tool for international benchmarking of patient experiences and satisfaction.

This study aimed to further validate the ECCQI in larger and more uniform groups of high volume tumours such as breast and prostate cancer. A second objective was the verification of the influence of cultural factors of the country to determine its possible use in international benchmarking.

**Methods:**

Data from two survey studies in eight European countries were combined. Socio-demographic correlations were analysed with Kruskall-Wallis and Mann-Whitney tests. Cronbach’s alpha was calculated to validate internal consistency. Influences of *masculinity* (MAS), *power distance* (PD) and *uncertainty avoidance* (UA) were determined by linear regression analysis in a general model and subgroup models.

**Results:**

A total of 1322 surveys were included in the analysis (1093 breast- and 348 prostate cancer patients). Cronbach’s alpha was good (α ≥ 0.7) or acceptable (0.5 ≤ α ≤ 0.7) in 8 out of 9 questionnaire categories, except in the category ‘Safety’ (α = 0.305). Overall ECCQI scores ranged from 22.1 to 25.1 between countries on a 1–35 scale (categories had a 1–4 scale). In certain subcategories such as ‘Organisation’ (range 2.2 vs 3.0) and ‘Supervision & Support’ (range 3.0 vs 3.8) a large difference was observed between countries. Differences in ‘Overall opinion’ were however small: mean scores of 3.7 vs 3.9, whereas median scores were all the maximum of 4.0. *Power distance* was positively associated with higher patient satisfaction scores whereas *Uncertainty avoidance* was negatively associated with these scores. *Masculinity* was only associated with patient satisfaction scores in lower educated patients. We found the highest impact of culture on overall scores in Hungary and Portugal and the lowest in Romania.

**Conclusions:**

The ECCQI shows high internal consistency in all categories except ‘Safety’. Especially in separate categories and overall ECCQI scores the questionnaire showed discriminative value. This study showed a positive correlation of *power distance* and a negative correlation for *uncertainty avoidance* in some countries. When using the ECCQI for international benchmarking these two dimensions of culture should be taken into account.

**Supplementary Information:**

The online version contains supplementary material available at 10.1186/s12885-021-07943-0.

## Background

Oncological care is complex and multifaceted. Patients often see multiple healthcare providers that are engaged in prevention, diagnosis, treatment and follow-up. This requires a high degree of coordination and if inadequately organised, can result in fragmented and discontinued care and bad experiences for patients [1]. Patient centeredness is defined as: care that respects and responds to individual patient’s preferences, needs, and values, and involves clinical decisions guided by patients. Improved health outcomes and better treatment adherence are associated with this approach [2, 3]. Wessels et al. [4] reported that expertise and attitude of healthcare providers were more important to cancer patients than healthcare professionals expected. This underlines the importance of patient reported information on the perspective of the patient, which can be obtained using questionnaires. A generic consumer quality index was developed and piloted in six European countries in a previous study, the European Cancer Consumer Quality Index (ECCQI) [5]. An advantage of a generic questionnaire is, the possibility of usage for patients irrespective of tumour types. Moreover, this enables international comparison and benchmarking of patient experiences [3].

The main objective of this study is to further validate the ECCQI in two large volume tumour patient groups, breast and prostate cancer patients, as a generic instrument applicable in an international setting. Breast and prostate cancer were selected as both are one of the top five cancers worldwide [6]. The International Agency for Research on Cancer (IARC) estimates that worldwide from 2020 to 2040 the incidence of breast cancer will increase with 40% (from 2.179.457 to 3.059.829 patients) and for prostate cancer with 69% (from 1.356.176 to 2.293.818 patients) [7].

The results of the pilot of the ECCQI by Wind et al. [5] demonstrated significant differences in patient satisfaction between countries. The (sub) groups in this pilot where however small and the influence of differences in country culture was not taken into account [5]. According to Napier et al. [8] culture should be considered when looking at health and healthcare provision, as social determinants of health can vary from culture to culture and cultural attitudes of both patients and providers can vary over place. For example variations in health between European countries could partly be explained by cultural differences [9]. Hofstede’s cultural six-dimension model [10] categorises aspects of cultural behaviour across countries so that they can be measured and compared [11]. An example of cultural attitudes influencing healthcare can be found by looking at *power distance*, one of the cultural dimensions of Hofstede [10]. In high *power distance* society’s hierarchy is important, patients will treat doctors as superiors. In contrast, in low *power distance* society’s patients treat doctors as equals [12]. *Uncertainty avoidance,* also part of Hofstede’s model, can also be used to explain differences when looking at health [10]. *Uncertainty-avoiding* cultures look for structure in their organisations, institutions and relations in order to make events clearly interpretable and predictable [10]. Self-ratings of health across countries tend to correlate negatively with the *uncertainty avoidance* index [13], in other words the higher the *uncertainty avoidance* the lower the rating of one owns health. Hofstede’s *masculinity* domain relates to ambition as a driving force and values being assertive and competitive compared to more *feminine* values like modesty and caring [12]. A *feminine* culture is focused more on quality of life and process versus a *masculine* culture with a stronger focus on task and more result orientation, e.g. is the patient cured [12]. Little is known about the link between patient perceived healthcare quality and cultural dimensions, hence the effect of cultural attitudes on patient reported experiences of care is unknown. Therefore, the second objective of this study is the verification of the influence of cultural factors in responses to this questionnaire in view of its possible use in international benchmarking.

## Methods

The questionnaire used for this study, the European Cancer Consumer Quality Index (ECCQI), was previously develop and piloted by Wind et al. [5]. Data of breast and prostate cancer patients from two studies, the study in which the ECCQI was piloted [5] and a study that focused on patient involvement in which the ECCQI was further validated, were combined i.e. participating patients of both studies were included in our analysis if they met the inclusion criteria. We applied the following inclusion criteria: (1) Respondents had to be at least 18 years old, (2) Respondents had to be examined, treated or had aftercare for cancer within the last 2 years in the examined institute, (3) Age, gender and level of education had to be known and (4) at least 50% of the questions had to be answered.

The data of the first (pilot) study [5] was collected in six hospitals in Hungary, Portugal, Romania, Lithuania and Italy (*n* = 2) via a paper based survey and through an online survey in The Netherlands in 2015. In the first study all hospitals collected data for both prostate cancer patients and breast cancer patients. Respondents were selected by convenience sampling. The data of the second (involvement) study was collected in two other Dutch hospitals (one hospital collected only breast cancer patients and one hospital collected only prostate cancer patients) and one Czech (both prostate cancer patients and breast cancer patients collected) and Finnish hospital (only breast cancer patients collected) via an online survey in 2019. In total patients from 11 hospitals in eight countries in total (Hungary, Portugal, Romania, Lithuania, Italy, The Netherlands, Czech Republic and Finland) were included based on both studies. Both in the online as in the paper based survey the option *Force answer* was not used.

### European Cancer consumer quality index

The ECCQI measures patient experiences and satisfaction with cancer care in hospitals in European countries. In the study by Wind et al. [5] patients indicated that in general the questionnaire was appropriate to measure patient satisfaction and experience. Confirmatory factor analysis revealed that the ECCQI measurement model had a moderate to good fit to the data in the first study [5] (RMSEA = 0.039, CFI = 0.943). The ECCQI consists of 63 questions divided into 11 categories. The core of the ECCQI measurement instrument are questions about the experiences of patients with care that are formulated as experience questions and questions about general appreciation. For the corresponding answer categories, the following applies:

- Experience questions are formulated in terms of how often a particular quality aspect occurred (e.g. ‘Did you understand the therapist’s explanation?); the corresponding answer categories are ‘never’, ‘sometimes’, ‘usually’ and ‘always’. If in questions of experience a frequency distribution is not relevant (a quality aspect is present or not) we worked with ‘no’, ‘yes’ as answer categories. For the questions pertaining to the attention of health professionals we had the following categories: ‘none of them’, ‘only nurses’, ‘only doctors’, only others’, ‘most’ and ‘all of them’.
- For general appreciation questions, the respondent is asked to indicate on a scale of 0 (very bad) to 10 (excellent) what he/she thinks of the care provider or aspects of the care provision.
  The three categories with demographic or disease specific information had different answer categories and were used as background so were not part of the analysis. The analysis therefore includes 9 categories (40 questions). These categories are: Organisation (5); Safety (2); Attitude of Healthcare Professionals (6); Communication and information (4); Own inputs (2); Coordination (4); Supervision and support (10); Rounding of treatment (5); Overall opinion (2). Patients were given the opportunity to comment on the questionnaire. The full questionnaire can be found in Additional file 1.

### Cultural influences

To adjust for cultural differences, we used Hofstede’s cultural six-dimension model. For our study we use the three commonly used cultural domains [14] of *masculinity* (MAS), *power distance* (PD) and *uncertainty avoidance* (UA) domains [15, 16]. Within Hofstede’s model each domain is rated on a 1–100 scale in which, i.e., a score of 100 on masculinity describes a highly masculine society, which is associated with lower and more extreme scores on reviews [10, 12].

MAS ranged between 14 (The Netherlands) and 88 (Hungary). PD ranged between 33 (Finland) and 90 (Romania). The lowest UA score was 53 (The Netherlands) and the highest UA was 99 (Portugal). A low masculine score indicates more tenderness and sympathy for others, possibly resulting in less willingness to provide criticism and therefore higher satisfaction scores. The used cultural model is a general description of a culture and does not have scores for socio-demographic groups. The effect of cultural differences was analysed using linear regression with MAS, PD and UA as independent variable for total scale score. The scores for MAS, PD and UA of all nationalities in the dataset were collected from ‘Hofstede’s insights country comparison tool’ on 6-12-2019 and can be found in Additional file 2.

### Recoding

Data were recoded in order to be analyzed. Almost all categories of the ECCQI consist of questions with four response options which were recoded into: never = 1, sometimes = 2, usually = 3 and always = 4. For the categories that did not consist of those four response options, the options were recoded into one of the four options above. For example, questions with sub-answers in the category “attitude of healthcare professional” had the option to state only nurses (score = 2), only doctors (score = 3) or only other healthcare professionals (score = 4) listen to the patient. These scores were recoded into “some of them did” (score = 2). The answer categories “most of them” (score = 5) and all of them (score = 6) were thereafter recoded into the scores 3 and 4. Questions related to the timing of events and the patients expectation, which have five answer categories, namely much sooner, sooner, when I’d expected it, later and much later were recoded into a four-scale by combining the options “sooner and much sooner” into one category, as done in the previous ECCQI study. Response codes of the questions about demographic characteristics were also recoded; (i) Age: 18–34, 35–64, and 65 or older; (ii) Years of education: low (1–8 years), moderate (9–13 years), and high (14 and higher). The answers ‘I don’t know/I no longer remember’ and ‘Not applicable’ were scored as missing.

### Analysis

Cases from the first and second study were selected from separate datasets and merged into one database if they met all four inclusion criteria. Data from this database was analysed in IBM SPSS statistics 24. Weighted means were calculated for each category of the ECCQI and country and depended on the number of items rated by the patient. Scale scores were summed and a weighted mean of overall patient experience was calculated [17]. The distribution of patient characteristics was determined by performing a chi-square test. Differences in total score were evaluated with a Kruskall-Wallis test and series of Mann-Withney tests, of which only the significant results are presented in this article. Internal consistency was evaluated using Chronbachs alpha for ordinal items [18]. The internal consistency was found good if α ≥ 0.7, acceptable if 0.5 ≤ α ≤ 0.7 and unacceptable if α ≤ 0.5 [19].

The influence of socio-demographic characteristics on the score of the ECCQI was analysed in order to verify whether age and education have an influence on reported quality. Gender was not analysed since no male breast cancer patients responded therefore analysing differences for tumour type is the same as analysing results for different gender. In addition, analysis of the dataset per participating county was performed, as previous results demonstrated significant differences between countries [5].

## Results

### Response

**Fig. 1:**
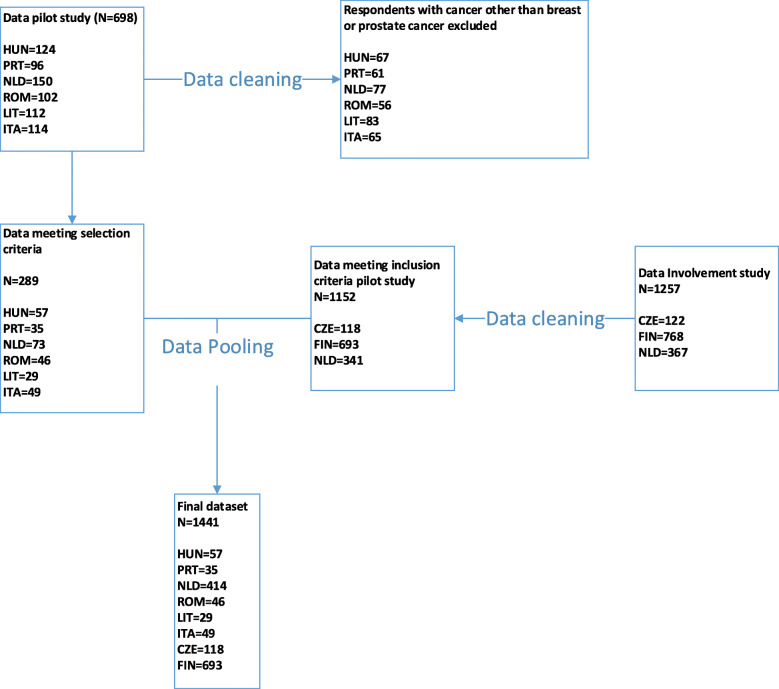
Flow-chart of survey inclusion

Pooling the datasets resulted in a final dataset of 1441 patients, of which 1093 breast cancer patients and 348 prostate cancer patients. The selection process of the surveys is visualised in Fig. 1 and respondent characteristics can be found in Table 1. Significant differences were found in the chi-square test for age (χ2(14) = 121.614, *p* < 0.000), sex (χ2(7) = 602.647, *p* < 0.000), education (χ2(14) = 452.345, *p* < 0.000) and physical health((χ2(28) = 118.856, *p* < 0.000) and last time patients went to the hospital (χ2(35) = 360.286, *p* < 0.000).

**Table 1:** ECCQI respondent characteristics

	HUN (*n* = 57)	PRT (*n* = 35)	NLD (*n* = 414)	ROM (*n* = 46)	LIT (*n* = 29)	ITA (*n* = 49)	FIN (*n* = 693)	CZE (*n* = 118)	Total (*n* = 1441)
**Age**									
18–34	–	–	1.0 (4)	4.3 (2)	6.9% (2)	4.1% (2)	1.4% (10)	5.1% (6)	1.8% (26)
35–64	68.4% (39)	60.0% (21)	42.3% (175)	84.8% (39)	72.4% (21)	81.6% (40)	63.3% (439)	73.7% (87)	59.8% (861)
≥ 65	31.6% (18)	40.0% (14)	56.8% (235)	10.9% (5)	20.7% (6)	14.3% (7)	35.2% (244)	21.2% (25)	38.4% (554)
**Tumour type**									
Prostate	12.3% (7)	22.9% (8)	63.3% (262)	15.2% (7)	44.8% (13)	8.2% (4)	–	39.8% (47)	24.1% (348)
Breast	87.7% (50)	77.1% (27)	36.7% (152)	84.8% (39)	55.2% (16)	91.8% (45)	100% (693)	60.2% (71)	75.9% (1094
**Education**									
Low	7.0% (4)	71.4% (25)	3.6% (15)	10.9% (5)	13.8% (4)	24.5% (12)	1.4% (10)	5.9% (7)	5.7% (82)
Moderate	43.9% (25)	17.1% (6)	33.8% (140)	37.0% (17)	31.0% (9)	46.9% (23)	63.3% (439)	34.7% (41)	48.6% (700)
High	49.1% (28)	11.4% (4)	62.6% (259)	52.2% (24)	55.2% (16)	28.6% (14)	35.2% (244)	59.3% (70)	45.7% (659)
**Physical health**									
Excellent	8.8% (5)	–	4.9% (20)	–	3.4% (1)	8.2% (4)	4.2% (29)	3.4% (4)	4.4% (63)
Very good	17.5% (10)	–	20.9% (85)	20.0% (9)	10.3% (3)	16.3% (8)	26.2% (181)	35.9% (42)	23.6% (338)
Good	28.1% (16)	34.0% (12)	58.7% (239)	51.1% (23)	55.2% (16)	42.9% (21)	48.1% (333)	46.2% (54)	49.9% (714)
Moderate	40.4% (23)	51.4% (18)	14.7% (60)	26.7% (12)	27.6% (8)	28.6% (14)	16.5% (114)	12.0% (14)	18.3% (262)
Poor	5.3% (3)	14.3% (5)	0.7% (3)	2.2% (1)	3.4% (1)	4.1% (2)	5.1% (35)	2.6% (3)	3.7% (53)
**Treatment stage**									
Test to ascertain diagnosis	1.8% (1)	–	5.1% (21)	2.2% (1)	17.2% (5)	–	0.9% (6)	0.8% (1)	2.4% (35)
Diagnosis known, will be treated soon	5.5% (3)	5.7% (2)	1.2% (5)	2.2% (1)	17.2% (5)	6.3% (3)	0.1% (1)	–	1.4% (20)
Curative treatment	32.7% (18)	57.1% (20)	16.3% (67)	65.2% (30)	44.8% (13)	70.8% (34)	24.8% (171)	21.2% (25)	26.4% (377)
No further treatment possible	–	–	0.5% (2)	2.2% (1)	–	–	0.1% (1)	–	0.3% (4)
Non-curative treatment	5.5% (3)	28.6% (10)	7.8% (32)	8.7% (4)	–	2.1% (1)	7.0% (48)	9.3% (11)	7.6% (109)
Check-up or treatments of the symptoms	43.6% (24)	8.6% (3) -	61.5% (252)	17.4% (8)	20.7% (6)	18.8% (9)	65.9% (455)	65.3% (77)	58.3% (834)
Finished check-ups and treatment	10.9% (6)		6.3% (26)	2.2% (1)	–	2.1% (1)	0.7% (5)	2.5% (3)	2.9% (42)
Patient does no longer remember	–	–	1.2% (5)	–	–	–	0.4% (3)	0.8% (1)	0.6% (9)
**Time since last hospital visit**									
< 1 month	54.4% (31)	91.4% (32)	38.5% (159)	73.9% (34)	50.0% (14)	83.7% (41)	28.8% (199)	42.4% (50)	38.9% (559)
1–2 months	17.5% (10)	2.9% (1)	24.0% (99)	6.5% (3)	14.3% (4)	6.1% (3)	8.8% (61)	40.7% (48)	15.9% (229)
2–4 months	10.5% (6)	2.9% (1)	22.3% (92)	13.0% (6)	21.4% (6)	–	16.5% (114)	12.7% (15)	16.7% (240)
4–8 months	5.3% (3)	–	9.2% (38)	4.3% (2)	3.6% (1)	4.1% (2)	20.8% (144)	3.4% (4)	13.5% (194)
8–12 months	7.0% (4)	2.9% (1)	3.6% (15)	2.2% (1)	7.1% (2)	4.1% (2)	19.2% (133)	–	11.0% (158)
> 12 months	5.3% (3)	–	2.4% (10)	–	3.6% (1)	2.0% (1)	5.8% (40)	0.8% (1)	3.9% (56)

Both percentages and absolute numbers are shown (between brackets)

### Results of the ECCQI per country

Table 2 shows the descriptive statistics of the ECCQI. The weighted mean of the scale scores ranged between 2.366 and 3.902. No significant differences were found in the initial comparison (χ2(7) = 31.226 *p* = 0.000) of the eight categories. Looking into the categories of the ECCQI we found that Italy scored high on the ‘Organisation of care’ (mean = 3.008, SD = 0.396). On the ‘Support and supervision’ category all countries scored above 3.0, except for Romania (mean = 2.959, SD = 0.661). Finland was found to be the highest scoring country in the category of ‘Rounding of the treatment’ (mean = 3.751, SD = 0.800). The highest scores of overall patient experiences were reported by patients from the Czech Republic (mean = 3.930, SD = 0.228) and Finland (mean = 3.907, SD = 0.314).

**Table 2:** Results per country

Nationality	Organisation	Safety	Attitude	Communication & Information	Own Input	Coordination	Supervision & Support	Rounding Treatment	Overall Opinion	Total
HUN										
Mean	2.875	3.802	3.353	3.474	3.333	3.468	3.336	2.882	3.722	23.773
Median	2.917	4.000	3.400	3.667	3.500	3.500	3.508	3.000	4.000	23.850
Range	1.833	1.500	2.200	2.250	2.500	1.500	2.111	1.800	1.000	29.972
Std. Deviation	0.496	0.358	0.571	0.568	0.733	0.365	0.533	0.561	0.384	5.119
PRT										
Mean	2.653	3.971	3.516	3.706	3.409	3.288	3.473	3.294	3.757	22.148
Median	2.750	4.000	3.500	3.875	3.750	3.250	3.600	3.292	4.000	23.125
Range	1.500	0.500	1.833	2.000	2.500	1.750	2.000	1.000	2.000	28.267
Std. Deviation	0.477	0.119	0.511	0.484	0.796	0.549	0.573	0.319	0.460	5.525
NLD										
Mean	2.222	3.872	3.526	3.650	3.667	3.335	3.338	3.176	3.836	24.153
Median	2.167	4.000	3.667	3.750	4.000	3.500	3.500	3.333	4.000	24.875
Range	2.470	2.500	2.333	2.920	3.000	2.750	3.000	2.470	3.000	29.930
Std. Deviation	0.520	0.381	0.507	0.478	0.577	0.597	0.698	0.493	0.411	4.759
ROM										
Mean	2.875	3.591	3.604	3.640	3.202	3.390	2.959	3.106	3.804	25.094
Median	3.000	4.000	3.800	3.750	3.000	3.500	3.111	3.417	4.000	24.992
Range	2.167	1.500	1.833	2.000	3.000	1.750	2.700	2.167	1.000	18.667
Std. Deviation	0.553	0.542	0.481	0.477	0.804	0.535	0.661	0.718	0.325	4.276
LIT										
Mean	2.817	3.648	3.660	3.43	3.396	3.471	3.473	3.282	3.793	23.511
Median	2.833	3.500	3.833	3.750	4.000	3.625	3.700	3.500	4.000	23.750
Range	1.000	1.000	2.000	2.500	3.000	2.500	1.900	2.067	2.000	18.600
Std. Deviation	0.329	0.362	0.533	0.597	0.978	0.567	0.633	0.572	0.491	4.889
ITA										
Mean	3.088	3.862	3.369	3.530	3.013	3.027	3.125	3.157	3.739	22.104
Median	3.167	4.000	3.500	3.500	3.000	3.250	3.200	3.292	4.000	22.900
Range	1.333	1.000	2.200	1.667	3.000	2.500	2.200	1.167	2.000	25.750
Std. Deviation	0.396	0.289	0.572	0.455	0.839	0.563	0.688	0.406	0.456	5.510
CZE										
Mean	2.242	3.379	3.335	3.642	3.271	3.402	3.480	3.144	3.930	23.163
Median	2.200	3.500	3.333	4.000	4.000	3.500	3.750	3.291	4.000	23.567
Range	1.700	3.000	2.830	3.250	3.000	2.500	2.890	2.000	1.000	20.830
Std. Deviation	0.414	0.435	0.572	0.546	0.934	0.594	0.645	0.485	0.228	4.212
FIN										
Mean	–	3.834	3.240	3.398	3.497	3.057	3.391	3.731	3.907	22.406
Median	–	4.000	3.333	3.500	3.500	3.000	3.600	3.833	4.000	22.333
Range	–	2.500	3.000	4.250	4.000	3.750	3.800	4.000	3.000	30.350
Std. Deviation	–	0.372	0.564	0.633	0.977	0.628	0.800	0.863	0.314	3.539
Total										
Mean	2.366	3.902	3.362	3.512	3.492	3.199	3.342	3.365	3.867	23.119
Median	2.333	4.000	3.500	3.750	4.000	3.250	3.500	3.400	4.000	23.083
Range	2.470	4.500	3.000	4.250	4.000	4.000	3.800	4.130	3.000	30.850
Std. Deviation	0.572	0.491	0.562	0.583	0.871	0.621	0.710	0.715	0.359	4.302

For all categories the minimum score is 1 and the maximum score is 4, except for total where the maximum is 35

A series of 28 Mann-Whithney tests, which were used as post-hoc analysis, demonstrated significant differences in mean total score between multiple comparisons of countries. Portugal’s score was significantly lower than the scores of The Netherlands (U = 5379.000, *p* = 0.011) and Romania (U = 545.000, *p* = 0.013). Portugal scored higher than Italy (U = 572.500, *p* = 0.010) and Czech Republic (U = 1483.500, *p* = 0.012). Finland scored lower than three countries, namely the Czech Republic (U = 33,824.000, *p* = 0.003), The Netherlands (U = 123,967.500, *p* = 0.000) and Romania (U = 12,821.000, *p* = 0.026). The last significant difference was a significantly higher score for Finland in the comparison with Italy (U = 13,179.000, *p* = 0.009).

The scale scores for each category are comparable to the scale scores between countries, which can be found in Table 2. We found that the category ‘Organisation’ was the category with the lowest average scale score. This category scored 2.3 on average, compared to average scale scores of > 3.0 in other categories of the ECCQI.

### Patient characteristics

Patients 65 and older reported a significantly higher score (mean = 3.469, SD = 0.389) compared to patients aged between 34 and 65 (mean = 3.520, SD = 0.381) (U = 218,217.000, *p* = 0.007). No significant differences in mean scale score were found between subgroups based on tumour type (equal to men and women), educational level and physical health.

### Internal consistency

Five categories of the ECCQI had a good internal consistency level (α > 0.7), namely ‘Attitude of the healthcare professional’, ‘Communication and information’, ‘Supervision and support’, ‘Rounding of the treatment’ and ‘Overall opinion’. The internal consistency of the categories ‘Coordination’, ‘Organisation’ and ‘Own inputs’ were acceptable (0.5 ≤ α ≤ 0.7). The internal consistency of ‘Safety’ was unacceptable (α = 0.305).

The category ‘Organisation’ with an overall acceptable internal consistency had unacceptable internal consistencies in the Czech Republic, Lithuania and Portugal and could not be calculated for Finland due to a large proportion of patients skipping questions of this category. The category ‘Safety’ had an unacceptable or barely acceptable internal consistency in most counties, and could not be calculated for Portugal due to zero variance in one of the two questions of this category. The only exception was seen in the Czech Republic, with α of 0.703. The overall internal consistency and internal consistencies per country can be found in Table 3*.*

**Table 3:** Cronbach’s alpha (*α)* for each ECCQI category per country

*Country*	*HUN*	*PRT*	*NLD*	*ROM*	*LIT*	*ITA*	*FIN*	*CZE*	*Total*
*Category (N Items)*									
Organisation (5)	**0.698** (21)	**0.347** (9)	**0.549** (216)	**0.668** (30)	**0.237** (11)	**0.631** (17)	–	**0.338** (25)	**0.621** (328)
Safety (2)	0.170 (48)	–	**0.440** (268)	**0.525** (43)	**0.089** (25)	**0.558** (47)	**0.224** (691)	**0.703** (101)	**0.312** (1255)
Attitude of HP (6)	**0.725** (28)	**0.848** (21)	**0.799** (3)	**0.897** (25)	**0.920** (9)	**0.864** (34)	**0.768** (680)	**0.835** (116)	**0.790** (1261)
Communication and information (4)	**0.830** (50)	**0.780** (26)	**0.760** (309)	**0.796** (43)	**0.802** (23)	**0.615** (41)	**0.686** (691)	**0.784** (101)	**0.723** (1283)
Own inputs (2)	**0.397** (40)	**0.775** (22)	**0.752** (299)	**0.679** (35)	**0.827** (14)	**0.687** (36)	**0.540** (690)	**0.819** (49)	**0.594** (1185)
Coordination (4)	**0.496** (49)	**0.638** (31)	**0.688** (258)	**0.514** (42)	**0.829** (25)	**0.706** (46)	**0.481** (691)	**0.721** (70)	**0.553** (1211)
Supervision and support (10)	**0.841** (18)	**0.856** (16)	**0.969** (17)	**0.929** (15)	**0.913** (9)	**0.923** (21)	**0.819** (125)	**0.770** (15)	**0.858** (236)
Rounding of treatment (5)	**0.775** (3)	**0.889** (4)	**0.843** (14)	**0.887** (10)	**0.698** (8)	**0.625** (4)	**0.724** (143)	**0.685** (17)	**0.740** (203)
Overall opinion (2)	**0.676** (54)	**0.851** (35)	**0.845** (402)	**0.546** (46)	**0.857** (29)	**0.849** (46)	**0.871** (691)	**0.749** (115)	**0.834** (1417)

The α for each ECCQI category per country is displayed in bold. For each α, the valid N is shown between brackets. Means α could not be calculated

### Influence of culture

Eight regression models for different socio-demographic groups were made using MAS, PD and UA as determinants based on the linear regression analysis. As none of the determinants had a significant influence on the youngest age group, no model for this group is included in this article. The constants of the seven models included in this article (see Table 4) varied between 24.983 (constant of breast model) and 28.608 (constant moderate education model). The maximum value of the intercept is 35 (maximum possible score on the ECCQI). MAS had no significant influence on total scale score. The coefficients for MAS were only significant in the models of the three different education groups. A negative association with MAS was found in the lower educated patient model (β = − 0.610, *p* = 0.014). A positive association was found in the models of moderate (β = 0.037, *p* = 0.031) and higher educated patients (β = 0.042, *p* = 0.023).

**Table 4:** Significant regression models for different sociodemographic groups with MAS, UA and PD as predictor

Model	Constant	Sign p	MAS β	Sign p	PD β	Sing p	UA β	sign p
Breast	24.983	0.000	0.016	0.100	0.115	0.000	− 0.113	0.000
Prostate	25.681	0.000	−0.031	0.189	0.013	0.829	−0.033	0.594
35–64	25.119	0.000	0.012	0.314	0.093	0.000	−0.097	0.002
> 65	25.376	0.000	−0.027	0.118	0.099	0.002	`-0.091	0.016
Low educated	26.723	0.000	−0.610	0.014	0.087	0.141	−0.069	0.167
Moderate educated	28.608	0.000	0.037	0.031	0.128	0.000	−0.192	0.000
High educated	28.124	0.000	0.042	0.023	0.129	0.000	−0.185	0.000

PD was significant positive in all models, varying between 0.093 and 0.129, except in the prostate cancer model. A high score on PD is therefore associated with a higher score on the ECCQI. UA had in almost all regressions a significant negative association with perceived quality of care, varying between − 0.091 and − 0.192. So the higher the score on UA the lower the score on the ECCQI would be. However, no significant influence of UA was found on the total scale score in the prostate cancer, and lower educated regression models.

## Discussion

In this study we further validated the ECCQI in eight countries among breast and prostate cancer patients. Comparability is an essential prerequisite of (international) benchmarking. It is therefore important to correct as much as possible for potential influencing factors [20]. We found differences between countries with both a small number of included as large number of included surveys on both the overall score and individual categories. Differences in score seem therefore to be independent from number of respondents, which enforces that applicability of the ECCQI for benchmarking. The significant differences ranged from 22.104 to 25.094 on a 1–35 scale. Patient experiences is on average scored the lowest in Italy and the highest in Romania. It must be considered that all Finnish respondents were breast cancer patients, in contrast to other countries, of which the respondent groups existed of both breast and prostate cancer patients. Although we found significant differences in mean scale scores, the total scale scores of the countries varied within a small range. It must be considered that all participating countries have a high level of quality of care since they are listed in the top 25 of the health access and quality index (HAQ). The HAQ ranks the access and quality of care of 195 countries [21].

The second objective, verifying the influence of cultural factors, helps to further establish the ECCQI as a tool for international benchmarking by looking into a possible influencing factor. We found that *masculinity* is negatively associated with reported patient experiences in lower educated patients. This could imply that the ECCQI score in masculine countries will be lower than in more feminine counties for lower educated patients. The opposite effect was found for patients with a moderate or higher education; these patients will probably score higher in a more masculine country. The effect of masculinity for lower educated patients on the patient reported experience is comparable to the association of lower patient satisfaction in masculine societies [15, 16]. *Uncertainty avoidance* is also negatively associated with patient reported experiences in this study. This is consistent with the theory that, although medical statistics show no evidence of objective health differences, people in uncertainty tolerant countries still feel healthier [10]. A positive association was found between the *power distance* and patient reported experience of care. This means that patient reported experience score is higher in societies with a large power distance. Our general model demonstrated that in case of equal scores on *power distance* and *uncertainty avoidance*, i.e. for Romania, the effects of these cultural determinants is eliminated. All else the same, the patient satisfaction score of countries with a *power distance* score higher than their *uncertainty avoidance* score will be affected positively. When the ECCQI is used for international benchmarking it is important to look into differences in the cultural domains and if applicable correct for them. However, to further determine the influence of cultural differences on satisfaction with healthcare it would be suggested to assess people from different cultural backgrounds within one country. This eliminates the influence of the healthcare system setting.

### Limitations

The internal consistency of the categories of the ECCQI was acceptable to good in most categories. Previous assumptions, that the low internal consistency of the category ‘Safety’ might have been caused by the low number of respondents, were falsified. A low internal consistency was namely also reported in countries with a high number of respondents. In addition, Cronbach’s alpha for Portugal could not be calculated since one of the two questions had no variance. The category ‘Safety’ in the ECCQI should therefore be re-examined. The category ‘Organisation’ for Finland could not be calculated due to too little answers. We did not include the option “*Force answer”* in our survey tool, as forcing an answer is associated with higher drop-out rates [22].

The results of this dataset may be influenced by the differences in response of the various participating countries. A possible limitation of this study design is the sampling method. With convenience sampling the chance of selection bias is high which could have influenced the outcomes. For example, with regard to education level a majority of the Portuguese patients had a low education level, a majority of the Italian patient had a moderate education while in the other countries the majority had a high education level. Regarding physical health, patients in Portugal were more negative giving a moderate score, while in the other countries most patients rated their physical health as good or excellent. Analysis of the total study population however showed no influence of demographic characteristics.

The response target of the studies of which we pooled data was both set on 100 per participating hospital per tumour type. The second study however exceeded this target by 2.65. It is likely that the high response is caused by the differences in recruitment strategy of the involvement study, in which hospitals sent the survey per email to patients. This difference in response between recruitment strategies was also found in the first study, in which the institutes using a digitalized strategy had a higher response rate than the centres using a paper based survey. At the start of the recruitment, the Czech hospital stated that it is likely that a low number of elderly patients would respond due to digital illiteracy. The proportion of elderly patients participating in the Czech Republic did however deviate little from the proportions of participating elderly patients in The Netherlands and Finland. We did encounter a big difference in response between breast cancer patients (75.9%) and prostate cancer patients (24.1%). A possible explanation for this could be that breast cancer has a higher incidence in Europe (female breast cancer is the most commonly diagnosed cancer, prostate cancer holds a third place) [23]. There were also more hospitals in this study that provided data for breast cancer patients (10 out of 11) compared to prostate cancer (9 out of 11).

None of the hospitals that participated in the first study participated again in the second study and there were 4 years between the first and the second study.

## Conclusion

This research confirmed conclusions about differences in patient satisfaction using the ECCQI as a measure. Significant differences between countries are likely not to be influenced by numbers of respondents. This study demonstrated good and acceptable internal consistency of the ECCQI. The items in the ‘Safety’ category of the ECCQI should be re-evaluated in order to hopefully increase the internal consistency of this category. Although the differences between the total scores are little, the ECCQI can discriminate between countries and used for benchmarking when looking into the category scores of this ECCQI. To our knowledge, this is the first study that reports associations of cultural aspects divided in *masculinity*, *power distance* and *uncertainty avoidance* with experiences and satisfaction of cancer patients measured by the ECCQI. The positive effect of *power distance* is however often outweighed by the negative effect of *uncertainty avoidance*. *Power distance* and *uncertainty avoidance* should therefore be included in international benchmarking in order to make valid comparisons.

## Supplementary Information

**Additional file 1.** Full ECCQI questionnaire.

**Additional file 2.** Scores for MAS, PD and UA of all nationalities in the dataset collected from ‘Hofstede’s insights country comparison tool’.

## Data Availability

The datasets supporting the conclusions of this article are included within the article (and its additional files). The full data set on the outcomes of the questionnaire are available upon request (please contact the corresponding author: ankewind@gmail.com).

## Abbreviations

CZE: Czech Republic
ECCQI: European cancer consumer quality index
FIN: Finland
HAQ: Health access and quality index
HUN: Hungary
IARC: International Agency for Research on Cancer
ITA: Italy
LIT: Lithuania
MAS: Masculinity
NLD: The Netherlands
PD: Power distance
PRT: Portugal
ROM: Romania
SD: Standard deviation
UA: Uncertainty avoidance
