# Supplementary material for: Validating a generic cancer consumer quality index in eight European countries, patient reported experiences and the influence of cultural differences
Source: BMC Cancer. 2021 Mar 6;21:231. doi: 10.1186/s12885-021-07943-0 (PMC7937284; doi:10.1186/s12885-021-07943-0)
Supplement: Supplementary file 1 — Additional file 1. Full ECCQI questionnaire. [file 12885_2021_7943_MOESM1_ESM.docx]

**THE ECCQI QUESTIONNAIRE
online survey**

**Introduction**

The purpose of this questionnaire is to measure the quality of care for cancer patients in the hospital as experienced by patients. Measuring the quality of care is part of a study on patient involvement in the design, evaluation and improvement process of cancer care pathways at the University of Twente. A care path describes how a patient goes through a certain care process and where and with whom there are decision moments in the care process.
It takes approximately 15 minutes to complete the questionnaire. Participation in this study is entirely voluntary. Participating or not participating has no influence whatsoever on your possible further treatment.

This questionnaire was sent by the hospital on behalf of the researchers. Therefore, after you have completed this questionnaire, the researchers only have access to the data that you have entered in this questionnaire. All your information is treated in strict confidence. This means that no one will ever know who gave which answers. Only the researchers have access to your information. The answers you have given to this questionnaire will be kept for 15 years and will be stored at the University of Twente.
If you want to participate in this research, fill in the question below and click on ">" at the bottom right of this page.

**Informed consent**

I have read and understood the text above. By answering "Yes" I give permission for anonymous processing of the answers I have given.

- Yes, I authorize the anonymous processing of my answers

**Instructions**

It is important that the questions are filled in by the person to whom the e-mail with link from this survey has been sent. It is not the intention to give the questionnaire to someone else. If this person is too ill to complete the questionnaire, we hope that someone can help you complete this questionnaire. This is also possible if that person does not understand the language in which this survey is written. The experience of the person addressed should be entered. It is possible that a certain question does not apply to you or that you have not (yet) experienced a certain part of care. Then answer these questions with "not applicable", "I do not know", or the additional answer option for the specific question.

Q1 Have you had any examinations, treatments and/or the first follow-up check for cancer in the hospital that approached you for participating in this questionnaire since 01-01-2018 and are you older than 18?

- No
- Yes

This questionnaire concerns the care you have had at the hospital since 01-01-2018. Please do not include any experiences you have had at other hospitals. We wish to know about your experiences since 01-01-2018, and not about experiences with any examinations, treatments, and aftercare you may have had before 01-01-2018

Q2 Which form of cancer do you have or have you had?

- Breast cancer
- Prostate cancer

Q3 In which month and year was the diagnosis made? 

________________________________________________________________

Q4 For which examinations and/or treatments have you been to this hospital since 01-01-2018?

- Examinations, e.g. physical examination, X-ray examination, ultrasound, puncture, blood test, CT scan, MRI scan, PET scan
- Operation
- Radiotherapy
- Chemotherapy
- Hormonal therapy
- Immunotherapy
- Aftercare
- Other treatment________________________________________________

Q5 Which of the following applies most to your current situation? (mark only one answer)

- I am having investigations in order to make a diagnosis
- I have been diagnosed and will be treated soon
- I am having treatment that is intended to cure
- The diagnosis has been made and can no longer be treated for my disease
- The treatment I am receiving is not intended to cure the tumor, but to control the symptoms associated with the disease and/or to slow down the growth of the tumor
- I have finished having treatment and attend this hospital for check-ups and/or for treatment of the symptoms associated with the disease
- I have finished having treatment and check-ups
- I no longer remember

Q6 When was the last time you were in the hospital for examinations, treatment (s) and / or checks for cancer?

- Less than 1 month ago
- 1-2 months ago
- 2-4 months ago
- 4-8 months ago
- 8-12 months ago
- Over 12 months ago

ORGANIZATION

Q7 Was your diagnosis of cancer made at this hospital?

- No
- Yes

Q8 How long did it last between your referral to the hospital and your first visit there?

- Less than 6 weekdays
- 6-10 weekdays
- 11-15 weekdays
- More than 15 weekdays
- I don't know/ I no longer remember
- Not applicable

Q9   How long did it last between your first visit/examination and your diagnosis?

- Less than 6 weekdays
- 6-10 weekdays
- 11-15 weekdays
- More than 15 weekdays
- I don't know/I no longer remember
- Not applicable

Q10 Did you hear the diagnosis sooner or later than you had expected?

- Much sooner
- Sooner
- When I'd expected it
- Later
- Much later
- I don't know/I no longer remember

Q11 Once the diagnosis was known, was it possible to start treatment as quickly as you wanted?

- Yes
- No
- I don't know/ I no longer remember
- Not applicable

Q12 To what extent have you experienced the waiting times as a problem (such as waiting times between examinations, treatments and results)

- As a large problem
- As a small problem
- It was not a problem

Q13  If you desired this, was it possible at this hospital to plan several appointments for examination and/or treatment on the same day?

- Never
- Sometimes
- Usually
- Always
- I do not know/ I no longer remember
- Not applicable

SAFETY

Q14 When you were being given medicine, did anyone check that it was really intended for you – by asking your name, for example, or checking your hospital wristband?

- Never
- Sometimes
- Usually
- Always
- I don't know/I no longer remember
- Not applicable, I did not take any medicine

Q15 Before treatment, examination or an operation began, did anyone check that you were the right person – by asking your name and date of birth, for example?

- Never
- Sometimes
- Usually
- Always
- I don't know/ I no longer remember

ATTITUDE OF HEALTHCARE PROFESSIONALS

Q16 Did the healthcare professionals listen to you attentively?

- None of them
- Only nurses
- Only doctors
- Only others, namely ________________________________________________
- Most
- All of them

Q17 Did the healthcare professionals have enough time for you?

- No, none of them did
- Only nurses
- Only doctors
- Only others, namely ________________________________________________
- Most of them did
- Yes, all of them did

Q18 Did the healthcare professional take you seriously?

- No, none of them did
- Only nurses
- Only doctors
- Only others, namely ________________________________________________
- Most of them did
- Yes, all of them did

Q19 Were there opportunities to talk with your healthcare professionals about how you felt?

- Never
- Only nurses
- Only doctors
- Only others, namely ________________________________________________
- Mostly
- Always

Q20 Were the healthcare providers in the hospital willing to talk with you about errors or issues that you do believe that did not go well?

- Never
- Sometimes
- Usually
- Always
- I don't know
- Not applicable

Q21 Did the healthcare professionals pay attention to your loved one(s)?

- None of them
- Only nurses
- Only doctors
- Only others, namely ________________________________________________
- Most
- All of them

Q22 Do your healthcare professionals show due respect to faith or philosophy of life?

- No, none of them
- Only nurses
- Only doctors
- Only others, namely ________________________________________________
- Most
- Yes, all of them

COMMUNICATION AND INFORMATION

Q23 Did healthcare professionals explain things to you in ways that were clear and understandable?

- Never
- Sometimes
- Usually
- Always

Q24 Did the healthcare professionals give you information about any side-effects of the treatment?

- Never
- Sometimes
- Usually
- Always
- I don't know/ I no longer remember
- Not applicable

Q25 During your treatment, were you informed about its effect (for example whether you were responding to it)?

- Never
- Sometimes
- Usually
- Always
- I don't know/I no longer remember
- Not applicable

Q27 Was the written information about the examinations or treatment clear?

- Never
- Sometimes
- Usually
- Always
- I don't know/ I no longer remember
- Not applicable (6)

OWN INPUTS

Q28 If you wanted, could you take part in decisions about the care and treatment you received?

- Never
- Sometimes
- Usually
- Always
- Not applicable, I didn't want to be

Q29  Was it possible for loved ones to be involved in discussions on your care and treatment?

- Never
- Sometimes
- Usually
- Always
- Not applicable

COORDINATION

Q30  Were the treatment and examinations you had from different healthcare professionals well coordinated?

- Never
- Sometimes
- Usually
- Always
- I don't know

Q31  Were your healthcare professionals aware of the appointments you had with other healthcare professionals?

- Never
- Sometimes
- Usually
- Always
- I don't know

Q32 Did you always deal with the same person in this hospital – such as a doctor or nurse – when anything needed to be arranged (planning and coordinating appointments, for example)?

- Never
- Sometimes
- Usually
- Always
- I don't know
- Not applicable

Q33 Were you seen by the same care providers during your investigations and treatments?

- Never
- Sometimes
- Usually
- Always
- I no longer remember
- Not applicable

SUPERVISION AND SUPPORT

Q34Did the caregivers take your pain complaints seriously?

- Yes
- No
- Not applicable, I had no complaints of pain

Q35 During the diagnostic phase, was attention paid to your pain?

- Never
- Sometimes
- Usually
- Always
- I don't know/I no longer remember

Q36 During the treatment phase, was attention paid to your pain?

- Never
- Sometimes
- Usually
- Always
- I don't know/ I do not remember

Q37 During aftercare, was attention paid to your pain?

- Never
- Sometimes
- Usually
- Always
- I don't know/I no longer remember

Q38 Did the healthcare professionals take complaints of fatigue seriously?

- Yes
- No
- Not applicable, I had no symptoms of fatigue

Q39 During the diagnostic phase, was attention paid to your complaints about fatigue?

- Never
- Sometimes
- Usually
- Always
- I don't know/I no longer remember

Q40 During the treatment phase, was attention paid to your complaints about fatigue?

- Never
- Sometimes
- Usually
- Always
- I don't know/I no longer remember

Q41 During the aftercare, was attention paid to your complaints about fatigue?

- Never
- Sometimes
- Usually
- Always
- I don't know/I no longer remember

Q42 Could you talk with healthcare professionals about how you felt?

- Never
- Sometimes
- Usually (only with nurses)
- Usually (doctors only)
- Usually with only others, namely:
- Always
- I don't know/I no longer remember
- Not applicable

Q43 Did this hospital provide you with information about help with coping with emotions and other forms of counselling on this?

- Never
- Sometimes
- Usually
- Always
- I don't know/I no longer remember
- Not applicable (6)

Q44 Did this hospital provide you with information about help with dealing with practical problems caused by cancer and other forms of counselling on this?

- Never
- Sometimes
- Usually
- Always
- I don't know/I no longer remember
- Not applicable

Q45 Did healthcare professionals inform you about patient organizations?

- Never
- Sometimes
- Usually
- Always
- I don't know/I no longer remember
- Not applicable

Q46 Was it possible to talk to a spiritual or moral counselor, such as a hospital chaplain or humanistic counselor?

- Never
- Sometimes
- Usually
- Always
- I don't know/I no longer remember
- Not applicable

ROUNDING OFF TREATMENT

Q47 Was your treatment concluded at the hospital?

- No
- Yes

Q48 When your treatment in this hospital was concluded, were you informed about possible symptoms or health problems you should be aware of/watch out for?

- No, not at all
- Not really
- More or less
- Yes, fully
- I don't know/I no longer remember
- Not applicable

Q49 Did you know who you could approach in this hospital with questions or problems after treatment had been concluded?

- No, not at all
- Not really
- More or less
- Yes, fully
- I don't know/I no longer remember
- Not applicable

Q50 Were important people and organizations, such as your general practitioner/family doctor, home care provider, rehabilitation center) informed that your hospital treatment had been concluded?

- No, not at all
- Not really
- More or less
- Yes, fully
- I don't know/I no longer remember
- Not applicable

Q51 Were the care and support you needed at home arranged for you?

- No ,not at all
- Not really
- More or less
- Yes, fully
- I don't know/I no longer remember
- Not applicable

Q52 Were you offered help with your questions about resuming your day-to-day activities (family, school, work) at the check-up?

- Never
- Sometimes
- Usually
- Always
- I don't know/I no longer remember
- Does not apply

OVERALL OPINION

Q53 Which score would you award this hospital?
 **0**means very bad indeed, 10 means excellent.

- 10
- 9
- 8
- 7
- 6
- 5
- 4
- 3
- 2
- 1
- 0

Q54 How likely is it that you would recommend the hospital to other patients with cancer?
a **0**means very unlikely, a **10**means very likely

- 10
- 9
- 8
- 7
- 6
- 5
- 4
- 3
- 2
- 1
- 0

Q55  How do you score the contribution of care to your quality of life?
A **0**means a very low contribution, a **10**means a very high contribution

- 10
- 9
- 8
- 7
- 6
- 5
- 4
- 3
- 2
- 1
- 0

Q56  Name one thing that should have been different about the care you received in the hospital

________________________________________________________________

ABOUT YOURSELF

Q57 What is your age

- 18-24
- 25-34
- 35-44
- 45-54
- 55-64
- 65-74
- 75 or more

Q58 Are you a male or female?

- Male
- Female

Q59 How many years of education have you attended? (including primary education, but without short courses)

________________________________________________________________

Q60 How would you describe your physical health in in general?

- Excellent
- Very good
- Good
- Moderate
- Poor

Q61 How would you  describe your mental health in general?

- Excellent
- Very good
- Good
- Moderate
- Poor

Q62 Has someone helped you to complete this questionnaire?

- No
- Yes

Q63 How did that person help you?

- By reading out the questions
- By selecting or writing down answers
- By answering the questions for me
- By translating the questions into my language
- By helping me in a different way, namely ________________________________________________
