# Supplementary material for: Validating a generic cancer consumer quality index in eight European countries, patient reported experiences and the influence of cultural differences
Source: BMC Cancer. 2021 Mar 6;21:231. doi: 10.1186/s12885-021-07943-0 (PMC7937284; doi:10.1186/s12885-021-07943-0)

Additional file 2

Scores for MAS, PD and UA of all nationalities in the dataset collected from ‘Hofstede’s insights country comparison tool’


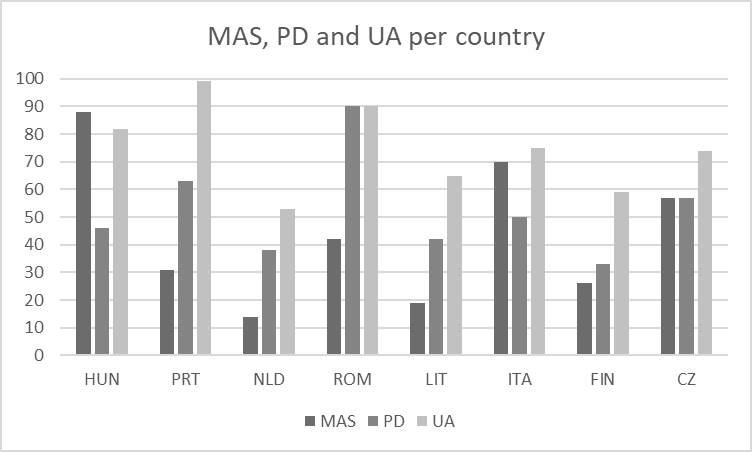

Supplement: Supplementary file 2 — Additional file 2. Scores for MAS, PD and UA of all nationalities in the dataset collected from ‘Hofstede’s insights country comparison tool’. [file 12885_2021_7943_MOESM2_ESM.docx]
